# Supplementation of β-mannanase alone or in combination with xylanase and β-glucanase enhanced growth performance, non-starch polysaccharide degradation, and gastrointestinal environment of broilers offered wheat-based diets

**DOI:** 10.1016/j.aninu.2025.05.008

**Published:** 2025-09-29

**Authors:** Eunjoo Kim, Mingan Choct, Anna Fickler, Guilherme A.M. Pasquali, Leon Hall, Tamsyn M. Crowley, Nishchal K. Sharma

**Affiliations:** a School of Environmental and Rural Science, University of New England, Armidale, NSW 2350, Australia; b BASF SE, Ludwigshafen 67056, Germany; c Poultry Hub Australia, University of New England, Armidale, NSW 2350, Australia

**Keywords:** Broiler, β-glucanase, β-mannanase, NSP-degrading enzyme, Non-starch polysaccharides, Xylanase

## Abstract

Nutritional strategies to enhance the utilization of undigested dietary components for sustainability and better production efficiency are constantly being explored in poultry. This study aimed to investigate the effects of β-mannanase alone or in combination with a xylanase and β-glucanase preparation on growth performance, nutrient and energy utilization, and gastrointestinal environment of broilers offered wheat-based diets. At d 0 post–hatch, 384 mixed-sex Cobb 500 broilers were assigned to 4 treatments in a 2 × 2 factorial arrangement, with β-mannanase (with or without) and xylanase + β-glucanase (XG, with or without) as the 2 factors. Each treatment contained 8 replicates of 12 birds per pen and 96 birds per treatment. During d 0 to 10, birds offered diets supplemented with XG (*P =* 0.023) or β-mannanase (*P =* 0.010) had higher weight gain, and β-mannanase supplementation improved (*P =* 0.040) feed conversion ratio (FCR) compared to the unsupplemented control. During d 0 to 35, XG increased (*P =* 0.035) and β-mannanase tended (*P =* 0.096) to increase the weight gain of birds. The combination of β-mannanase and XG interacted (*P =* 0.016) for FCR where either XG or β-mannanase alone improved FCR compared to the unsupplemented control but there were no further improvements when they were added in tandem. At d 21, XG decreased (*P <* 0.001) ileal digesta viscosity, and XG (*P <* 0.001) or β-mannanase (*P =* 0.004) decreased ileal pH. β-Mannanase increased total tract retention of dry matter (DM, *P =* 0.001), nitrogen (N, *P =* 0.019), soluble non-starch polysaccharides (NSP, *P =* 0.020), insoluble NSP (*P <* 0.001), total NSP (*P <* 0.001), free oligosaccharides (*P =* 0.023), calcium (Ca, *P <* 0.001), and showed tendency (*P =* 0.098) to increase nitrogen-corrected apparent metabolizable energy (AMEn). Dietary XG increased apparent total tract retention of DM (*P =* 0.017), soluble NSP (*P <* 0.001), Ca (*P =* 0.005), apparent metabolizable energy (AME) (*P =* 0.024), and AMEn (*P =* 0.020). β-Mannanase and XG combination showed a tendency (*P =* 0.073) to interact for AME such that the highest AME was observed in this group. β-Mannanase and XG combination interacted (*P =* 0.034) for butyric acid concentration in the caecal contents where β-mannanase or XG alone increased the concentration of butyric acid compared to the unsupplemented control which was further enhanced when they were added in tandem. β-Mannanase (*P =* 0.049) or XG (*P =* 0.046) increased total short-chain fatty acid concentration in the caecal contents. These findings suggest that dietary supplementation of β-mannanase or XG alone provided comparable growth performance benefits and positively affected the gut environment, however, their combination further enhanced energy utilization and butyric acid production, and the latter may have implications in broiler's gut health and immune response.

## 1 Introduction

The poultry industry is moving towards more sustainable production with a minimal environmental footprint. One way of achieving sustainability is to produce feed with higher utilization of nutrients in the bird and minimal wastage to the environment. With this broad objective in mind, Kim et al. (2022a) characterized the undigested components of major nutrients throughout the gastrointestinal tract of broilers on a wheat-based diet and found that around 32% of the nutrients remained undigested at the total tract (excreta) level at 35 d of age. Around one-third of these undigested nutrients were non-starch polysaccharides (NSP) followed by protein and other nutrients. These undigested nutrients significantly affect production efficiency, profitability, litter quality, shed environment, and sustainability of poultry production. Hence, there is an opportunity to develop novel targeted approaches to better utilize the undigested dietary components for sustainable poultry production.

Commercial broiler diets contain 10% to 12% total NSP, of which 1% to 2.5% is water soluble (Bach Knudsen, 1997). The NSP content in poultry feed in Australia is largely contributed by wheat and soybean meal as these are the 2 commonly used ingredients. The concentration of soluble and insoluble arabinoxylans in wheat has been reported to be in the range of 9 to 18 g/kg and 50 to 63 g/kg dry matter (DM), respectively (Bach Knudsen, 1997; Choct, 2006; Cowieson et al., 2015). High molecular weight soluble NSP can increase the viscosity of the intestinal contents (Choct and Annison, 1990) and negatively affect bird growth as the viscous gut environment hinders nutrient digestion and absorption by interfering with the accessibility of endogenous enzymes to their substrates (Choct and Annison, 1992; Bedford, 1997). Dietary insoluble NSP, on the other hand, acts as a physical barrier during digestion (Hetland et al., 2004) entrapping nutrients within the cell wall matrix, thereby decreasing the nutritional value of feedstuffs (Choct, 2015). Although a small proportion of NSP is fermented by gut microbiota in the distal ileum and caeca, birds lack endogenous enzymes for the effective breakdown of NSP in feed and thus exogenous carbohydrases such as xylanase and β-glucanase are routinely added in broiler diets. However, due to the variety of NSP present in plant materials, xylanase alone or in combination with β-glucanase, may not be effective in digesting all the NSP found in feed. Vegetable proteins, such as soybean meal (SBM), contains around 35% carbohydrates including 10% free sugars (5% sucrose, 4% stachyose and 1% raffinose), <1% starch and between 20% and 30% NSP. The NSP in SBM contains 8% cellulose and the remaining are pectic polysaccharides and oligosaccharides (Choct et al., 2010). The pectic polysaccharides refer to rhamnogalacturonans, and the common structural feature is α-1,4-linked D-galacturonic acid and α-1,2-L-rhamnose units, as well as a large number of neutral sugars attached as side chains including L-arabinose, D-galactose, D-xylose, and lesser amounts of L-fucose and D-glucuronic acid (Choct, 1997). Data on the presence of specific mannans in commonly used feed ingredients is scarce, despite frequent inferences about their occurrence. Indeed, mannose, the monomeric constituent of mannans, is present in many feed ingredients albeit in small quantities (Bach Knudsen, 1997). For instance, soybean contains between 1% and 2% of mannose, which is the building block of mannans. Although mannan structures or their precursors are speculated to be present in the cotyledons of soybeans due to observed mannanase activity in the endosperm (Rueckel et al., 2024), to the best of the authors’ knowledge, the presence of mannans has only been demonstrated in soybean hulls (Whistler and Saarnio, 1957), not in the cotyledon of soybean. However, reports on varying levels of mannans in SBM are widespread. For instance, Hsiao et al. (2006) reported mannan contents of 1.26% in SBM derived from dehulled soy and 1.61% in SBM from non-dehulled soy. It appears that these researchers extrapolated the levels of beta-mannans from the amounts of mannose without fully elucidating the polymeric structure(s) in which the mannose existed.

One of the hypotheses regarding the benefits of mannanase supplementation is that dietary mannans could be partially degraded by mannanase, leading to the release of mannan-oligosaccharides (MOS) in the digestive tract. Kalidas et al. (2017) isolated MOS from palm kernel cake and reported a significant increase in the growth of *Lactobacillus reuteri* C1 strain in vitro - known to be a beneficial organism. A recent meta-analysis (Kiarie et al., 2021) reported significant improvements in weight gain, feed conversion ratio (FCR), and nitrogen-corrected apparent metabolizable energy (AMEn) of broilers offered diets supplemented with β-mannanase. However, these studies were based on corn-soy diets with or without corn distillers’ dried grain with solubles (DDGS) and guar meal. Also, most of these studies had a basal diet (control) without phytase limiting our understanding of the added value of β-mannanase or xylanase + β-glucanase (XG) in broilers. Thus, there is a dearth of knowledge on the impact of β-mannanase supplementation, either alone or in combination with XG, in wheat-soy based diets containing phytase. Such information is highly useful in countries like Europe and Australia where wheat is a major energy source. We hypothesized that supplementing β-mannanase in a wheat-soy based diet containing phytase improves growth performance and increases the retention of nutrients, energy, and NSP, which are further enhanced when supplemented in combination with XG. This study aimed to investigate the effects of β-mannanase alone or in combination with XG on growth performance, nutrient and energy utilization, NSP degradation, and gastrointestinal tract environment of broilers offered wheat-based diets.

## 2 Materials and methods

### 2.1 Animal ethics statement

This experiment was authorized to be conducted under the supervision of the Animal Ethics Committee of the University of New England (UNE), Australia (Approval No. ARA22 to 043). All broiler management procedures including health care, husbandry, and use of animals fulfilled the requirements of the Australian Code for the Care and Use of Animals for Scientific Purposes (NHMRC, 2013).

### 2.2 Housing and bird management

A total of 384 mixed-sex Cobb 500 broilers were obtained from the Baiada hatchery in Tamworth, New South Wales, Australia. On hatch day (d 0), the birds were randomly distributed into 32-floor pens of equal size in an environment-controlled Rob Cumming Poultry Innovation Centre at UNE. Each pen contained a feeder and 2 nipple drinkers providing the birds with ad libitum access to feed and water. The pens were spread with hardwood shavings to a depth of around 7 cm. The room temperature was controlled, starting at 32 to 33 °C and gradually lowered to 22 °C by d 21, where it remained until d 35. The lighting schedule began with 23 h of light at approximately 40 lux on d 1, with one additional hour of darkness introduced daily until 6 h of darkness was achieved. After that, the birds were kept under 18 h of light per day at 10 lux for the rest of the study.

### 2.3 Diets and experimental design

The diets were thoroughly mixed and cold pelleted at 65 °C, and the starter diets were crumbled. The basal diet consisted of wheat and soybean meal as major ingredients and were analyzed for CP, and amino acids (AA) by near-infrared spectroscopy (Foss NIR 6500, Foss A/S, Hillerød, Denmark) standardized to Evonik AMINONIR Advanced calibration before formulation. The ingredient and nutrient composition of the basal diet are presented in Table 1. The basal diet contained phytase (Natuphos E, BASF SE Animal Nutrition, Ludwigshafen, Germany) at 1000 FTU/kg with matrix values for energy, amino acids, and minerals. The diets were formulated to meet the Cobb 500 nutrient specifications (Cobb-Vantress, 2022). Vitamin and mineral premixes were included in the diets to meet the requirements, following the manufacturer's guidelines (UNE VM and UNE TM, Rabar Pty Ltd., Moama NSW, Australia). Titanium dioxide was incorporated at 0.5% in the grower diet as an indigestible marker. A starter crumble was provided for the first 10 d post–hatch. The treatment grower and finisher diets were then offered as 3-mm pellets from d 10 to 21 and d 21 to 35, respectively.

**Table 1:** Ingredients and nutrient composition of the basal diet.

Item	Starter	Grower	Finisher
**Ingredients, % (as-is)**			
Wheat	56.46	57.66	59.72
Wheat bran	5.00	7.50	10.00
Soybean meal	32.20	29.00	25.30
Canola oil	2.90	2.80	2.70
L-Lys·HCl	0.03	0.01	–
D,L-Met	0.22	0.14	0.15
L-Thr	0.06	0.03	–
Choline chloride 60%	0.07	0.06	0.06
Vitamin premix^1^	0.09	0.09	0.09
Mineral premix^2^	0.11	0.11	0.11
Sand	0.42	0.42	0.42
Titanium dioxide	–	0.50	–
Limestone	0.82	1.09	1.05
NaCl	0.35	0.35	0.32
Dicalcium phosphate	1.26	0.23	0.07
Phytase^3^	0.01	0.01	0.01
Total	100.00	100.00	100.00
**Calculated values, % (as-is)**			
DM	90.9	90.8	90.7
AME_n_, kcal/kg	3084	3080	3094
Dig Lys	1.13	1.04	0.95
Dig Met	0.51	0.42	0.42
Dig Met + Cys	0.85	0.75	0.74
Dig Thr	0.77	0.70	0.63
Dig Val	0.98	0.93	0.88
Dig Iso	0.89	0.84	0.79
Dig Arg	1.43	1.35	1.26
Dig Trp	0.28	0.27	0.25
Ca	0.96	0.80	0.74
Available P	0.48	0.40	0.37
Sodium	0.18	0.18	0.16
Choline, mg/kg	1700	1600	1550
**Analyzed values, % (DM basis)**			
GE, kcal/kg	3800	3920	3944
CP	21.4	20.1	19.6
Ca	1.48	0.89	0.84
P	1.11	0.73	0.69
Soluble NSP	–	1.3	–
Insoluble NSP	–	8.9	–
Total NSP	–	10.2	–
Free oligosaccharides	–	5.1	–

DM = dry matter; AMEn = nitrogen-corrected apparent metabolizable energy; Ca = calcium; P = phosphorus; NSP = non-starch polysaccharides.

1 Vitamin premix per kilogram diet (UNE VM, Rabar Pty Ltd, Moama NSW, Australia): vitamin A, 12 MIU; vitamin D, 5 MIU; vitamin E, 75 mg; vitamin K, 3 mg; nicotinic acid, 55 mg; pantothenic acid, 13 mg; folic acid, 2 mg; riboflavin, 8 mg; cyanocobalamin, 0.016 mg; biotin, 0.25 mg; pyridoxine, 5 mg; thiamine, 3 mg; antioxidant, 50 mg.

2 Mineral premix per kilogram diet (UNE TM, Rabar Pty Ltd, Moama NSW, Australia): Cu, 16 mg, as copper sulfate; Mn, 60 mg, as manganese sulfate; Mn, 60 mg, as manganous oxide; I, 0.125 mg, as potassium iodide; Se, 0.3 mg; Fe, 40 mg, as iron sulfate; Zn, 50 mg, as zinc oxide; Zn, 50 mg, as zinc sulfate.

3 Natuphos E (BASF SE Animal Nutrition, Ludwigshafen, Germany).

All birds were weighed on arrival and assigned into 4 treatments in a 2 × 2 factorial arrangement, with β-mannanase (with or without) and XG (with or without) as the 2 factors. The treatments were: 1) a basal diet with phytase at 1000 FTU/kg (unsupplemented control), and the basal diet supplemented with 2) β-mannanase (Natupulse TS, 800 TMU/kg), 3) XG (Natugrain TS, 560 TXU/kg and 250 TGU/kg of xylanase and β-glucanase, respectively), and 4) a combination of β-mannanase and XG. These doses represent the commercial recommendations (BASF SE Animal Nutrition, Ludwigshafen, Germany). One thermostable mannanase unit (TMU) is defined as the amount of enzyme that produces reducing carbohydrates having a reducing power corresponding to 1 μmol mannose from locust bean gum (0.3 g/100 mL buffer solution) in 1 min under the assay conditions of 50.0 ± 0.1 °C and pH 3.5. One thermostable endo-xylanase unit (TXU) is defined as the amount of enzyme that released 5 μmol reducing sugars, measured as xylose equivalents per minute from a buffer solution containing 1 g arabinoxylan per 100 mL at pH 3.5 and 40 °C. One thermostable endo-glucanase unit (TGU) is defined as the amount of enzyme that released 1 μmol of reducing sugars, measured as glucose equivalents per minute from a buffer solution containing 0.714 g β-glucan per 100 mL at pH 3.5 and 40 °C. Each treatment contained 8 replicates of 12 birds per pen. The feed supplement was added at the expense of sand.

### 2.4 Growth performance

Birds and feed were weighed upon arrival and on d 10, 21, and 35 for each pen, while mortality was monitored and recorded daily. Feed intake, weight gain, and mortality-adjusted FCR were calculated for the different experimental periods.

### 2.5 Apparent total tract retention of nutrients and metabolizable energy

On d 15, four birds per pen (6 replicates/treatment) were transferred to cages and acclimatized for 2 d. Fresh water and feed were provided to all the birds ad libitum. From d 17 to 20, feed intake and total excreta output were measured quantitatively per cage daily for 3 consecutive days for determinations of apparent metabolizable energy (AME), AME_n_, apparent total tract retentions of DM, nitrogen (N), calcium (Ca), phosphorus (P), soluble NSP, insoluble NSP, and free oligosaccharides. At the end of the collection period, several sub-samples were taken from the total excreta and thoroughly homogenized. A 250 g representative sample was then placed in a plastic container, and stored at −20 °C until further processing.

### 2.6 Apparent ileal digestibility of nutrients, intestinal viscosity, and pH

On d 21, caged birds were euthanized by electrical stunning (MEFE CAT 44N, Mitchell Engineering Food Equipment, Clontarf, Queensland, Australia) and cervical dislocation. The carcass was dissected for the measurement of gut pH and collection of gut contents for measurements of viscosity, apparent ileal digestibility of DM and CP, and caecal short-chain fatty acids (SCFA).

The pH of the gizzard, jejunum, and ileal contents were determined in situ by inserting a pH probe (Sensorex, Garden Grove, CA, USA), connected to a pH meter (Mettler-Toledo Safeline Limited, Salford, UK), directly into the digesta ensuring the probe did not touch the intestinal wall. The procedure was repeated twice and an average pH value of the 2 readings was taken. The pH probe was rinsed with ultra-pure water between samples. Ileal digesta viscosity was measured in triplicate for each cage. The jejunal, ileal, and caecal contents were pooled per cage. Ileal digesta samples were added into 2 mL Eppendorf tubes, and the tubes were centrifuged at 10,000 × *g* for 10 min at room temperature. Viscosity was measured in 0.5 mL of the resulting supernatant, using a Brookfield DV3T Rheometer (AMETEK Brookfield, Middleboro, MA, USA), with CPA-40Z Spindle, at 25 °C. Viscosity measurements were expressed in centipoise (cPs) unit (1 cPs = 1/100 dyne s per cm^2^ = 1 mPa∙s) prior to statistical analysis. The remaining ileal and caecal contents were stored in a freezer at −20 °C until further processing.

### 2.7 Caecal short-chain fatty acids (SCFA)

Short-chain fatty acids and lactic acid concentrations were measured in the caecal digesta samples using the previously described method (Jensen et al., 1995) with some modifications. In brief, 1 mL of internal standard (0.01 mol/L ethylbutyric acid) was added to approximately 2 g of fresh digesta sample, mixed, and centrifuged at 3900 × *g* at 5 °C for 20 min. Approximately 1 mL of the resulting supernatant, 0.5 mL of concentrated HCl (36%), and 2.5 mL of ether were combined. The mixture was then centrifuged at 2000 × *g* at 5 °C for 15 min and 400 μL of the resulting supernatant was combined with 40 mL of N-tert-butyldi-methlsilyl-N-methyltrifuoroacetamide (MTBSTFA) in a gas chromatography (GC) vial. The samples were then heated at 80 °C for 20 min, left at room temperature for 48 h, and were analyzed on a Varian CP3400 CX gas chromatograph (Varian Analytical Instruments, Palo Alto, CA, USA). The SCFA concentration was expressed as μmol/g digesta.

### 2.8 Measurements and chemical analyses

The frozen excreta and ileal digesta samples were freeze-dried (Christ Alpha 1-4 LDplus, Osterode am Harz, Germany) and ground to pass through a 0.5-mm screen. Ground feed, excreta, and ileal contents were analyzed in duplicates for N by the Dumas method (method 968.06) of AOAC (1990) using a LECO FP-2000 automatic N analyzer (Leco Corporation, St. Joseph, MI, USA). Titanium dioxide was measured in duplicate for diets and digesta samples by UV-spectroscopy at 410 nm (Cary 50 Bio UV–Visible spectrophotometer equipped with a Cary 50 MPR microplate reader, Varian Inc., Palo Alto, CA, USA), using the method described by Short et al. (1996). The ground feed and freeze-dried samples were oven-dried to measure DM using the method 934.01 (AOAC, 1990). The gross energy (GE) of dried feed and excreta was determined using an adiabatic bomb calorimeter (IKA Werke, C7000, GMBH and Co., Staufen, Germany) with benzoic acid as the standard. Soluble and insoluble NSP and free oligosaccharide composition of feed and excreta samples (d 21) were determined following the procedure of Englyst et al. (1994) with some modifications as described by Theander et al. (1995) and Morgan et al. (2019) using Agilent 8890GC equipped with Agilent 7693A autosampler (Agilent Technologies Inc., Palo Alto, CA, USA).The X-ray fluorescence spectroscopy analysis was conducted using the Olympus Vanta M Series (Olympus Scientific Solutions Americas, Waltham, MA, USA) to detect calcium and phosphorus in the feed and excreta samples, with 1 g of sample placed in polyethylene XRF sample cups. The standard scan method used was the Geochem 3-beam method with 30 s scan per beam and 90 s in total (Vanta User's Manual, 2025).

The AME, apparent total tract retention and apparent ileal digestibility of nutrients were calculated by using the following equations:

$$\text{AME}=\frac{\left(\text{Feed}\;\text{intake}\times \text{GE}\;\text{diet}\right)-\left(\text{Excreta}\;\text{output}\times \text{GE}\;\text{excreta}\right)}{\text{Feed}\;\text{intake}}.$$

The AME_n_ was determined by correction for zero N retention by simple multiplication with 8.22 kcal/g N retained in the body.

$$\text{Apparent}\;\text{total}\;\text{tract}\;\text{retention}\;\text{of}\;\text{nutrient}\;\left(\%\right)=\frac{[\left(\text{Feed}\;\text{intake}\times \text{Nutrient}\;\text{in}\;\text{diet}\right)-\left(\text{Excreta}\;\text{output}\times \text{Nutrient}\;\text{in}\;\text{excreta}\right)}{\left(\text{Feed}\;\text{intake}\times \text{Nutrient}\;\text{in}\;\text{diet}\right)]}\times 100\text{;}$$

Apparent ileal digestibility of nutrient (%) = (Titanium in diet/Titanium in digesta × Nutrient in digesta/Nutrient in diet) × 100.

### 2.9 Statistical analysis

The data were analyzed by General Linear Model procedures of IBM SPSS statistical software version 28. Pen represented the replicate unit for statistical analysis. Data were tested for normal distribution using the Kolmogorov–Smirnov test and analyzed as a two-way ANOVA to assess the main effects of XG, β-mannanase, and their interaction. The statistical model used was as follows:

$$Y_{ij}=\mu +M_{i}+X_{j}+R_{ij}+\varepsilon_{ij}\text{,}$$

where *Y*_*ij*_ is the observation of dependent variables; μ is the overall mean; *M*_*i*_ is the β-mannanase effect; *X*_*j*_ is the XG effect, *R*_*ij*_ represents the interactive effect between the 2 factors, and *ε*_*ij*_ is the residual error for the observation.

The sex ratio of each pen was applied as a covariate in the analysis. When a significant difference was detected, means were separated by Tukey's HSD. Data that were not normally distributed were analyzed by using the Kruskal–Wallis test. Statistical significance was declared at *P <* 0.05 and a tendency to be significant was determined at 0.05 ≤ *P <* 0.10.

## 3 Results

### 3.1 Growth performance

The effect of in-feed carbohydrases on the growth performance of broilers is presented in Table 2. During d 0 to 10, birds fed a diet supplemented with XG (*P =* 0.023) or β-mannanase (*P =* 0.010) had higher weight gain compared to the non-supplemented control. During d 10 to 21 and d 21 to 35, there was no significant effect (*P >* 0.05) of XG, β-mannanase, or their combination on weight gain. During d 0 to 35, XG increased (*P =* 0.035) weight gain, and β-mannanase tended (*P =* 0.096) to increase the weight gain of birds. Dietary supplementation of XG or β-mannanase had no effect (*P >* 0.05) on feed intake of birds during d 0 to 10 and d 10 to 21. During d 21 to 35, β-mannanase and XG interacted (*P* = 0.029) for feed intake where, compared to the unsupplemented control, β-mannanase decreased feed intake of birds in the absence of XG but not in the presence of XG. β-Mannanase and XG tended (*P =* 0.061) to interact for feed intake during d 0 to 35 where β-mannanase supplementation resulted in the lowest feed intake in birds among all the treatments but was increased when combined with XG. β-Mannanase supplementation improved FCR during d 0 to 10 (*P =* 0.040) and d 21 to 35 (*P =* 0.002). The supplementation of XG improved FCR from 10 to 21 d (*P =* 0.012). β-Mannanase and XG interacted (*P =* 0.016) for FCR during d 0 to 35 where either XG or β-mannanase alone improved FCR compared to the unsupplemented group but there were no further improvements in FCR when they were added in tandem. Mortality was <4% and not affected by dietary treatment (data not shown).

**Table 2:** Effect of in-feed carbohydrases on growth performance of broilers^1^.

Item		Weight gain, g/bird				Feed intake, g/bird				Feed conversion ratio, g/g			
		0–10 d	10–21 d	21–35 d	0–35 d	0–10 d	10–21 d	21–35 d	0–35 d	0–10 d	10–21 d	21–35 d	0–35 d
XG	β-Mannanase												
–	–	237	725	1286	2247	290	982	2065^a^	3336	1.222	1.357^a^	1.604	1.482^a^
–	+	255	745	1281	2280	290	973	1886^b^	3150	1.139	1.309^b^	1.471	1.381^b^
+	–	253	753	1286	2292	292	957	1948^a^	3197	1.153	1.249^c^	1.516	1.394^b^
+	+	257	737	1355	2349	290	958	1997^a^	3244	1.133	1.298^b^	1.474	1.380^b^
SEM		2.2	5.4	13.6	14.5	2.7	9.4	26.1	30.6	0.1262	0.1271	0.1533	0.1052
Main effect													
XG	–	246	735	1283	2264	290	978	1976	3243	1.181	1.333	1.538	1.431
	+	255	745	1320	2321	291	957	1972	3221	1.143	1.273	1.495	1.387
β-Mannanase	–	245	739	1286	2270	291	969	2006	3266	1.188	1.303	1.560	1.438
	+	256	741	1318	2315	290	966	1942	3197	1.136	1.303	1.472	1.380
*P*-value													
XG		0.023	0.706	0.125	0.035	0.856	0.821	0.943	0.693	0.121	0.012	0.107	0.009
β-Mannanase		0.010	0.624	0.190	0.096	0.866	0.386	0.180	0.237	0.040	0.999	0.002	0.001
XG × β-mannanase		0.093	0.468	0.145	0.663	0.793	0.738	0.029	0.061	0.223	0.049	0.104	0.016

SEM = standard error of the mean.

1 XG, a xylanase and β-glucanase preparation (Natugrain TS); β-mannanase (Natupulse TS). Means within columns not sharing a common superscript are significantly different (*P <* 0.05).

### 3.2 Intestinal viscosity and pH

The effects of in-feed carbohydrases on intestinal viscosity and pH of broilers on d 21 are presented in Table 3. Dietary supplementation of XG decreased (*P <* 0.001) ileal digesta viscosity. β-Mannanase (*P =* 0.004) or XG (*P <* 0.001) supplementation decreased ileal pH. There was no effect (*P >* 0.05) of XG or β-mannanase on gizzard and jejunal pH.

**Table 3:** Effects of in-feed carbohydrases on intestinal viscosity and pH of broilers on d 21^1^.

Item		Viscosity, mPa·s		pH		
		Jejunum	Ileum	Gizzard	Jejunum	Ileum
XG	β-Mannanase					
–	–	2.28	3.30	3.32	6.01	6.59
–	+	2.38	3.27	3.17	5.95	6.42
+	–	2.08	2.50	3.33	6.06	6.38
+	+	2.34	2.70	3.32	5.98	6.20
SEM		0.147	0.172	0.075	0.050	0.053
Main effect						
XG	–	2.33	3.29	3.25	5.98	6.51
	+	2.21	2.60	3.32	6.02	6.29
β-Mannanase	–	2.18	2.90	3.32	6.04	6.49
	+	2.36	2.98	3.25	5.97	6.12
*P*-value						
XG		0.298	<0.001	0.370	0.486	<0.001
β-Mannanase		0.083	0.641	0.272	0.176	0.004
XG × β-mannanase		0.240	0.519	0.498	0.832	0.926

SEM = standard error of the mean.

1 XG, a xylanase and β-glucanase preparation (Natugrain TS); β-mannanase (Natupulse TS). Means within columns not sharing a common superscript are significantly different (*P <* 0.05).

### 3.3 Apparent ileal digestibility and total tract retention of nutrients, and apparent metabolizable energy

The effects of in-feed carbohydrases on apparent ileal digestibility, total tract retention of nutrients, and metabolizable energy (ME) of broilers are presented in Table 4. At d 21, there was no effect *(P >* 0.05) of XG or β-mannanase supplementation on apparent ileal digestibility of N. β-Mannanase supplementation increased apparent ileal digestibility of DM (*P =* 0.046), apparent total tract retention of DM (*P =* 0.001), N (*P =* 0.019), soluble NSP (*P =* 0.020), insoluble NSP (*P <* 0.001), total NSP (*P <* 0.001), free oligosaccharides (*P =* 0.023), Ca (*P <* 0.001), and showed a tendency (*P =* 0.098) to increase AME_n_. Dietary supplementation of XG increased apparent total tract retention of DM (*P =* 0.017), soluble NSP (*P <* 0.001), Ca (*P =* 0.005), AME (*P =* 0.024), AME_n_ (*P =* 0.020), and decreased (*P =* 0.027) apparent total tract retention of insoluble NSP. β-Mannanase and XG interacted (*P <* 0.001) for apparent total tract retention of P where β-mannanase or XG alone increased P retention compared to the unsupplemented group but there were no further improvements when they were added in tandem. β-Mannanase and XG showed a tendency (*P =* 0.073) to interact for AME and the highest AME was observed in the group supplemented with the combination of β-mannanase and XG.

**Table 4:** Effects of in-feed carbohydrases on apparent ileal digestibility, total tract retention of nutrients, and metabolizable energy of broilers (DM basis)^1^.

Item		Apparent ileal digestibility, %		Apparent total tract retention, % of intake								AME, kcal/kg	AME_n_, kcal/kg
		DM	N	DM	N	Soluble NSP	Insoluble NSP	Total NSP	Free oligosaccharides	Ca	P		
XG	β-Mannanase												
–	–	64.0	78.4	68.5	58.4	−1.0	24.8	21.5	65.0	52.6	53.5^c^	3292	3186
–	+	65.8	78.2	70.1	60.4	9.0	33.7	30.6	71.6	60.8	69.1^a^	3310	3198
+	–	65.0	79.9	69.8	58.5	21.1	18.5	18.9	67.6	58.6	60.8^b^	3313	3207
+	+	66.8	79.0	70.5	59.8	40.0	29.5	31.2	72.0	62.4	56.4^b^	3339	3232
SEM		0.92	1.41	0.30	0.44	5.71	2.19	2.40	2.04	1.21	1.80	10.0	10.7
Main effect													
XG	–	64.9	78.3	69.3	59.4	4.0	29.2	26.1	68.3	56.7	61.3	3301	3192
	+	65.9	79.4	70.1	59.1	30.6	24.0	25.0	69.8	60.5	58.6	3326	3219
β-Mannanase	–	64.5	79.2	69.1	58.5	10.0	21.7	20.2	66.3	55.6	57.1	3303	3196
	+	66.3	78.6	70.3	60.1	24.5	31.6	30.9	71.8	61.6	62.7	3324	3215
*P*-value													
XG		0.665	0.444	0.017	0.664	<0.001	0.027	0.672	0.564	0.005	0.150	0.024	0.020
β-Mannanase		0.046	0.683	0.001	0.019	0.020	<0.001	<0.001	0.023	<0.001	0.006	0.184	0.098
XG × β-mannanase		0.200	0.788	0.132	0.584	0.447	0.637	0.522	0.136	0.088	<0.001	0.073	0.575

DM = dry matter; N = nitrogen; NSP = non-starch polysaccharides; Ca = calcium; P = phosphorus; AME = apparent metabolizable energy; AME_n_ = nitrogen-corrected apparent metabolizable energy; SEM = standard error of mean.

1 XG, a xylanase and β-glucanase preparation (Natugrain TS); β-mannanase (Natupulse TS). Means within columns not sharing a common superscript are significantly different (*P <* 0.05).

### 3.4 Caecal short-chain fatty acids and lactic acid

The effect of in-feed carbohydrases on the caecal SCFA and lactic acid profile of broilers on d 21 is presented in Table 5. Dietary supplementation of XG increased (*P =* 0.045) and that of β-mannanase showed a tendency (*P =* 0.090) to increase acetic acid concentrations in the caecal contents. β-Mannanase and XG interacted (*P =* 0.034) for butyric acid concentration in the caecal contents where XG or β-mannanase alone increased the concentration of butyric acid which was further enhanced when they were added in tandem. β-Mannanase (*P =* 0.049) or XG (*P =* 0.046) supplementation increased total SCFA concentration in the caecal contents. There was no effect (*P >* 0.05) of XG or β-mannanase on the concentrations of formic, propionic, isobutyric, valeric, isovaleric, and lactic acid in the caeca.

**Table 5:** Effect of in-feed carbohydrases on caecal short-chain fatty acids and lactic acid concentrations of broilers on d 21 (μmol/g)^1^.

Item		Formic	Acetic	Propionic	Butyric	Isobutyric	Valeric	Isovaleric	Total SCFA	Lactic acid
XG	β-mannanase									
–	–	0.73	74.5	3.5	17.7^c^	0.41	0.50	0.13	97.5	0.37
–	+	0.41	80.2	4.5	21.3^b^	0.50	0.65	0.14	107.6	0.19
+	–	0.28	82.1	3.4	20.9^b^	0.43	0.60	0.13	107.9	0.63
+	+	0.22	95.7	5.1	24.0^a^	0.47	0.76	0.09	126.3	1.08
SEM		0.215	5.41	0.82	1.52	0.095	0.133	0.029	6.83	0.263
Main effect										
XG	–	0.57	77.4	4.0	19.5	0.45	0.57	0.13	102.6	0.28
	+	0.25	88.9	4.2	22.5	0.45	0.68	0.11	117.1	0.85
β-Mannanase	–	0.51	78.3	3.4	19.3	0.42	0.55	0.13	102.7	0.50
	+	0.32	88.0	4.8	22.6	0.48	0.70	0.11	117.0	0.63
*P*-value										
XG		0.525	0.045	0.758	0.043	0.959	0.312	0.482	0.046	0.112
β-Mannanase		0.707	0.090	0.116	0.033	0.535	0.184	0.554	0.049	0.885
XG × β-mannanase		0.893	0.477	0.636	0.034	0.782	0.425	0.355	0.546	0.245

SCFA = short-chain fatty acids; SEM = standard error of mean.

1 XG, a xylanase and β-glucanase preparation (Natugrain TS); β-mannanase (Natupulse TS). Means within columns not sharing a common superscript are significantly different (*P <* 0.05).

## 4 Discussion

Nutritional strategies to enhance the utilization of undigested dietary components for better production efficiency, profitability, and sustainability of poultry production are constantly being explored. In this study, birds in the control group excreted 31.5% DM at 21 d of age which is similar to that reported by Kim et al. (2022a) of 34% and 32% at 12 and 35 d of age, which highlights that younger birds excrete slightly more nutrients than the older. In this study, β-mannanase either alone or in combination with XG was added in feed as a strategy to enhance nutrient and energy utilization, NSP degradation, and growth performance of broilers. While NSP such as arabinoxylans and β-glucans present in feed can be hydrolyzed by the exogenous XG, β-mannans present in the ingredients, especially soybean meal, are not hydrolyzed by these enzymes due to their substrate specificity. Yet, nutritionists rarely use enzymes to target β-mannans in feed, as a low level of dietary β-mannans is thought to have negligible effects in poultry. Literature suggests that the antinutritive effects of β-mannans may be alleviated by supplementing the diets with β-mannanase, resulting in significant improvements in growth performance (Shastak et al., 2015). Most of the previous studies on β-mannanase were performed in broilers fed corn-soy based diets or diets containing alternative ingredients that had high β-mannan content. To date, the effects of β-mannanase either alone or in combination with XG on a wheat-soy based diet have not been thoroughly investigated in broilers.

In this study, dietary XG supplementation decreased ileal digesta viscosity and increased total tract retention of soluble NSP. This was expected and illustrates that a significant proportion of soluble polymeric arabinoxylans of wheat was hydrolyzed into more readily fermentable substrates, such as oligosaccharides, for hindgut microbiota. Dietary XG supplementation also improved the weight gain and FCR of birds. The mechanisms by which these enzymes exert positive effects on growth performance have been comprehensively studied in the past. These include digesta viscosity reduction, plant cell wall destruction resulting in higher retentions of nutrients, efficient energy utilisation, and in situ production of prebiotic oligomers, such as arabinoxylo-oligosaccharides, leading to increased caecal fermentation capacity. Such properties were evidenced in the current study. The increases in AME and AME_n_ from XG supplementation in the current study were 25 and 27 kcal/kg, which is slightly lower than the normal range of 30 to 150 kcal/kg increase (Adeola and Cowieson, 2011) and indicates that superior quality (e.g. high ME) of wheat and soybean meal was used in this experiment. This is supported by the observation that the analyzed AME value of the basal diet was higher than the calculated AME in the feed formulation. Yet, the combination of β-mannanase and XG improved energy utilisation of birds by leading to the highest AME which was 47 kcal/kg higher than the unsupplemented group, highlighting the benefits of adding β-mannanase in combination with XG in a wheat-soy-based diet.

Dietary supplementation of β-mannanase alone displayed similar growth performance benefits as that of XG but the improvement in FCR by β-mannanase was largely due to the reduced feed intake, especially during the finishing period, unlike XG where the improvement in FCR was largely driven by the increased weight gain. Interestingly, there was an interaction between the 2 enzymes for FCR where each enzyme alone improved FCR but there were no further improvements when they were added in tandem. This indicates that a maximum feed efficiency as a result of substrate degradation in the gut could have been achieved with individual enzyme supplementation. β-Mannanase or XG alone increased weight gain by 45 and 57 g, respectively during 0 to 35 d of age. The fact that β-mannanase or XG supplementation alone resulted in comparable improvements in weight gain and FCR highlights that the hydrolysis of relatively smaller quantities of β-mannans, as compared to larger quantities of arabinoxylans, yielded similar responses in terms of growth performance. β-Mannanase also increased total tract retentions of DM, N, Ca and P and showed a trend to increase AME_n_. Some of these benefits were also observed in previous studies where β-mannanase was supplemented in broiler diets based on wheat (Barekatain et al., 2024) and corn (Ferreira et al., 2016; Sacakli et al., 2024) and support our findings. Viscosity reduction has been proposed as one of the modes of action contributing to the beneficial effects of β-mannanase on growth performance and nutrient digestibility in poultry (Shastak et al., 2015). There is a clear effect of XG on reducing ileal digesta viscosity in the current study, demonstrating that arabinoxylans present in wheat are the major driver for creating a viscous gut environment. The earlier findings (Lee et al., 2003; Shastak et al., 2015; Latham et al., 2018) indicated that dietary β-mannanase effects on intestinal digesta viscosity are evident only when diets that are high in β-mannan or galactomannan content, which is the case when high β-mannan-rich ingredients such as guar meal is used. As soy mannans do not play a significant role in increasing intestinal viscosity in poultry compared to the mannans in guar meal (Gullon et al., 2009), β-mannanase supplementation likely presented a negligible impact on the gut digesta viscosity in the current study. β-Mannanase, however, increased the total tract retention of free-oligosaccharides, soluble NSP, insoluble NSP and total NSP. These findings corroborate the findings of Kim et al. (2022b) who reported reduced ileal soluble and insoluble NSP levels in birds fed wheat-based diet supplemented with β-mannanase. The higher retention of soluble and insoluble NSP with β-mannanase supplementation indicates an effective hydrolysis of β-mannans, leading to improved caecal fermentation to produce higher contents of butyric acid, acetic acid, and SCFA as observed in the current study. Increased retention of total NSP was achieved by β-mannanase supplementation which again highlights the benefits of adding β-mannanase in a wheat-based diet.

Endo-β-mannanases randomly cleave the backbone of β-mannans (Schroder et al., 2009) that are found in the hulls fraction and cell walls of soybean meal. This action releases proteins that are embedded within the cell wall matrix, making them available for further digestion (Shastak et al., 2015; Rueckel et al., 2024). A subtle disturbance in the cell wall structure by carbohydrases can also increase cell wall permeability, facilitating further degradation by the gut microbiota. The observed increased retentions of DM, N, Ca and P through β-mannanase supplementation in the present study likely result from the mannanolytic activity, which disrupts cell wall polysaccharides and provides birds with more available nutrients. Hence, the hydrolysis of β-mannans by endo-acting β-mannanases consequently releases manno-oligosaccharides (MOS) that have beneficial prebiotic effects (Rueckel et al., 2024). Such effects were evidenced by increased butyric acid, acetic acid, and total SCFA concentrations in the caeca in the current study. This action is similar to the soluble arabinoxylans breakdown by xylanase to release lower molecular weight oligosaccharides, which are selectively fermented by beneficial gut bacteria in the gut to produce SCFA (Courtin et al., 2008; Morgan et al., 2017; Kim et al., 2022c). Similar to the above findings, XG supplementation in the current study increased acetic acid, butyric acid, and total SCFA concentrations in the caeca. Indeed, β-mannanase and XG interacted such that their combination produced more butyric acid in the caecal contents compared to when they were added individually to the diet. The additive effect between β-mannanase and XG on butyric acid can be attributed to the fact that the oligosaccharides derived from arabinoxylans and mannans are distinct and may be utilized by different bacterial groups, as indicated by Figueiredo et al. (2020) and Asano et al. (2001). In situ production of butyric acid can improve intestinal epithelial integrity by fuelling epithelial cells, exert anti-inflammatory properties, and increase the metabolic activity of the intestinal microbiota (Guilloteau et al., 2010). A lower ileal pH with XG or β-mannanase supplementation in the current study is also indicative of increased SCFA production following fibre or oligosaccharides fermentation in the hindgut.

Increased butyric acid, increased total SCFA and a tendency to increase acetic acid concentrations in the caeca, and decreased ileal pH observed in the current study highlights alternate modes of action of β-mannanase. Increasing evidence suggests that β-mannanase may favourably modulate gut health, immune response, and microbiota in broilers (Bortoluzzi et al., 2019; Kiarie et al., 2021; Barekatain et al., 2024; Zhang et al., 2024), and these may be linked to increased butyric acid and SCFA production as observed in the current study. Some mannans comprise surface components of numerous pathogens, and as such β-mannan can be recognized by the host immune system as “Pathogen Associated Molecular Patterns” through several cell surface receptors in the gastrointestinal tract, consequently stimulating innate immune responses and negatively affecting the growth performance of birds (Dale et al., 2008; Arsenault et al., 2017). This feed-induced immune response can cost energy to the birds due to the activation of the immune system. The management of immune response by dietary β-mannanase addition was demonstrated in recent studies (Zhang et al., 2024; Barekatain et al., 2024). In this study, the beneficial effects of β-mannanase on growth performance were not related to intestinal viscosity reduction but rather improved nutrients and energy utilization, along with prebiotic effects as evidenced by increased caecal SCFA production and lower ileal pH possibly due to successful hydrolysis of dietary mannans. These changes in the gut environment possibly improved gut health and reduced feed-induced immune response in broilers.

## 5 Conclusion

This experiment demonstrated that around 31.5% of nutrients remained undigested and excreted by birds at 21 d of age and the application of NSP-degrading enzymes reduced the amount of excreted nutrients. The supplementation of either XG or β-mannanase alone in a wheat-soy based diet containing phytase led to comparable growth performance benefits and positively affected the gut environment. Although the synergistic effects of their combination were not significant for growth performance, their combination further enhanced energy utilization and butyric acid production. This may have implications in gut health and immune response in broilers. Further research is warranted to explore the benefits of dietary β-mannanase on intestinal function, intestinal inflammation and immunity in broilers fed wheat-soy based diets.

## Credit Author Statement

**Eunjoo Kim:** Writing – review & editing, Investigation, Data curation, Methodology, Formal analysis, Conceptualization. **Mingan Choct:** Supervision, Funding acquisition, Writing – review & editing, Methodology, Conceptualization. **Anna Fickler:** Methodology, Writing – review & editing. **Guilherme A.M. Pasquali:** Methodology, Writing – review & editing. **Leon Hall:** Writing – review & editing, Methodology, Project administration. **Tamsyn M. Crowley:** Resources, Writing – review & editing, Project administration. **Nishchal K. Sharma:** Writing – original draft, Methodology, Writing – review & editing, Project administration.

## Declaration of competing interests

Anna Fickler, Guilherme A.M.Pasquali and LeonHall are currently employed by BASF SE Animal Nutrition, Ludwigshafen, Germany. All authors hereby declare that they have no known conflicts of interest to disclose in relation to the work presented herein.

This study is funded by BASF SE Animal Nutrition, Ludwigshafen, Germany. Any financial or material support provided by BASF SE Animal Nutrition for the development of this work is solely for operational and research purposes, with no stipulations that would compromise the objectivity, integrity, or independence of the authors’ contributions.

## References

[bib1] Adeola O., Cowieson A.J. (2011). Board-invited review: opportunities and challenges in using exogenous enzymes to improve nonruminant animal production. J Anim Sci.

[bib2] AOAC (1990).

[bib3] Arsenault R.J., Lee J.T., Latham R., Carter B., Kogut M.H. (2017). Changes in immune and metabolic gut response in broilers fed β-mannanase in β-mannan-containing diets. Poult Sci.

[bib4] Asano I., Nakamura Y., Hoshino H., Aoki K., Fujii S., Imura N., Iino H. (2001). Use of mannooligosaccharides from coffee mannan by intestinal bacteria. Jpn Soc Biosci Biotechnol Agrochem.

[bib5] Bach Knudsen E.B. (1997). Carbohydrate and lignin contents of plant materials used in animal feeding. Anim Feed Sci Technol.

[bib6] Barekatain R., Hall L., Chrystal P.V., Fickler A. (2024). Nutrient utilisation and growth performance of broiler chickens fed standard or moderately reduced dietary protein diets with and without β-mannanase supplementation. Anim Nutr.

[bib7] Bedford M.R. (1997).

[bib8] Bortoluzzi C., Scapini L.B., Ribeiro M.V., Pivetta M.R., Buzim R., Fernandes J.I.M. (2019). Effects of β-mannanase supplementation on the intestinal microbiota composition of broiler chickens challenged with a coccidiosis vaccine. Livest Sci.

[bib9] Choct M., Annison G. (1990). Anti-nutritive activity of wheat pentosans in broiler diets. Br Poult Sci.

[bib10] Choct M. (1997). Feed non-starch polysaccharides: chemical structures and nutritional significance. Feed Mill Intern..

[bib11] Choct M., Annison G. (1992). Anti-nutritive effect of wheat pentosans in broiler chickens: roles of viscosity and gut microflora. Br Poult Sci.

[bib12] Choct M. (2006). Enzymes for the feed industry: past, present and future. Worlds Poult Sci J.

[bib13] Choct M., Dersjant-Li Y., McLeish J., Peisker M. (2010). Soy oligosaccharides and soluble non-starch polysaccharides: a review of digestion, nutritive and anti-nutritive effects in pigs and poultry. AJAS (Asian-Australas J Anim Sci).

[bib14] Choct M. (2015).

[bib15] Cobb-Vantress (2022). https://www.cobbgenetics.com/assets/Cobb-Files/2022-Cobb500-Broiler-Performance-Nutrition-Supplement.pdf.

[bib16] Courtin C.M., Broekaert W.F., Swennen K., Lescroart O., Onagbesan O., Buyse J., Decuypere E., Van de Wiele T., Marzorati M., Verstraete W., Huyghebaert G., Delcour J.A. (2008). Dietary inclusion of wheat bran arabinoxylooligosaccharides induces beneficial nutritional effects in chickens. Cereal Chem.

[bib17] Cowieson A.J., Schliffka W., Knap I., Roos F.F., Schoop R., Wilson J.W. (2015). Meta-analysis of effect of a mono-component xylanase on the nutritional value of wheat supplemented with exogenous phytase for broiler chickens. Anim Prod Sci.

[bib18] Dale N.M., Anderson D.M., Hsiao H. (2008). Identification of an inflammatory compound for chicks in soybean meal. Poult Sci.

[bib19] de Figueiredo F.C., de Barros Ranke F.F., de Oliva-Neto P. (2020). Evaluation of xylooligosaccharides and fructooligosaccharides on digestive enzymes hydrolysis and as a nutrient for different probiotics and Salmonella typhimurium. LWT.

[bib20] Englyst H.N., Quigley M.E., Hudson G.J. (1994). Determination of dietary fibre as non-starch polysaccharides with gas–liquid chromatographic, high-performance liquid chromatographic or spectrophotometric measurement of constituent sugars. Analyst.

[bib21] Ferreira H.C., Hannas M.I., Albino L.F.T., Rostagno H.S., Neme R., Faria B.D., Xavier M.L., Rennó L.N. (2016). Effect of the addition of β-mannanase on the performance, metabolizable energy, amino acid digestibility coefficients, and immune functions of broilers fed different nutritional levels. Poult Sci.

[bib22] Guilloteau P., Martin L., Eeckhaut V., Ducatelle R., Zabielski R., Van Immerseel F. (2010). From the gut to the peripheral tissues: the multiple effects of butyrate. Nutr Res Rev.

[bib23] Gullon P., Gullon B., Moure A., Alonso J., Dominguez H., Parajo J. (2009). Biological properties of mannans and mannan-derived products. Prebiot Probiot Sci Technol.

[bib24] Hetland H., Choct M., Svihus B. (2004). Role of insoluble non-starch polysaccharides in poultry nutrition. Worlds Poult Sci J.

[bib25] Hsiao H.Y., Anderson D.M., Dale N.M. (2006). Levels of β-mannan in soybean meal. Poult Sci.

[bib26] Jensen M.T., Cox R.P., Jensen B.B. (1995). Microbial production of skatole in the hind gut of pigs given different diets and its relation to skatole deposition in backfat. Anim Sci.

[bib27] Kalidas N.R., Saminathan M., Ismail I.S., Abas F., Maity P., Islam S.S., Manshoor N., Shaari K. (2017). Structural characterization and evaluation of prebiotic activity of oil palm kernel cake mannanoligosaccharides. Food Chem.

[bib28] Kiarie E.G., Steelman S., Martinez M., Livingston K. (2021). Significance of single β-mannanase supplementation on performance and energy utilization in broiler chickens, laying hens, turkeys, sows, and nursery-finish pigs: a meta-analysis and systematic review. Transl Anim Sci.

[bib29] Kim E., Morgan N.K., Moss A.F., Li L., Ader P., Choct M. (2022). Characterisation of undigested components throughout the gastrointestinal tract of broiler chickens fed either a wheat-or maize-based diet. Anim Nutr.

[bib30] Kim E., Moss A.F., Morgan N.K., Gharib-Naseri K., Ader P., Choct M. (2022). Ileal profile of non-starch polysaccharides and oligosaccharides in response to exogenous enzymes in broiler chickens offered wheat-or maize-based diets under subclinical necrotic enteritis challenge. Anim Nutr.

[bib31] Kim E., Moss A.F., Morgan N.K., Gharib-Naseri K., Ader P., Choct M. (2022). Non-starch polysaccharide-degrading enzymes may improve performance when included in wheat- but not maize-based diets fed to broiler chickens under subclinical necrotic enteritis challenge. Anim Nutr.

[bib32] Latham R.E., Williams M.P., Walters H.G., Carter B., Lee J.T. (2018). Efficacy of β-mannanase on broiler growth performance and energy utilization in the presence of increasing dietary galactomannan. Poult Sci.

[bib33] Lee J.T., Bailey C.A., Cartwright A.L. (2003). β-Mannanase ameliorates viscosity-associated depression of growth in broiler chickens fed guar germ and hull fractions. Poult Sci.

[bib34] Morgan N.K., Wallace A., Bedford M.R., Choct M. (2017). Efficiency of xylanases from families 10 and 11 in production of xylo-oligosaccharides from wheat arabinoxylans. Carbohydr Polym.

[bib35] Morgan N.K., Keerqin C., Wallace A., Wu S.-B., Choct M. (2019). Effect of arabinoxylo-oligosaccharides and arabinoxylans on net energy and nutrient utilization in broilers. Anim Nutr.

[bib36] NHMRC (2013). 8th ed. The National Health and Medical Research Council.

[bib38] Rueckel M., Janson S., Solbak A., Fickler A. (2024). Spatial activity mapping of ß-mannanase on soybean seeds. Sci Rep.

[bib39] Sacakli P., Ramay M.S., Ahsan U., Gebes E.S., Harijaona J.A., Fickler A., Shastak Y., Calik A. (2024). Effect of dietary β-mannanase supplementation on growth performance and nutrient retention in broiler chickens fed corn-soybean meal-based diets with low energy and amino acid density. Poult Sci.

[bib40] Schröder R., Atkinson R.G., Redgwell R.J. (2009). Re-interpreting the role of endo-beta-mannanases as mannan endotransglycosylase/hydrolases in the plant cell wall. Ann Bot.

[bib41] Shastak Y., Ader P., Feuerstein D., Ruehle R., Matuschek M. (2015). ß-Mannan and mannanase in poultry nutrition. Worlds Poult Sci J.

[bib42] Short F.J., Gorton P., Wiseman J., Boorman K.N. (1996). Determination of titanium dioxide added as an inert marker in chicken digestibility studies. Anim Feed Sci Technol.

[bib43] Theander O., Aman P., Westerlund E., Andersson R., Pettersson D. (1995). Total dietary fiber determined as neutral sugar residues, uronic acid residues, and Klason lignin (the Uppsala method): collaborative study. J AOAC Int.

[bib44] Vanta User’s manual (2025). https://hotrobotics.co.uk/wp-content/uploads/2021/07/Manual-DMTA-10072-01EN-Vanta-User-International.pdf.

[bib45] Whistler R.L., Saarnio J. (1957). Galactomannan from soy bean hulls. J Am Chem Soc.

[bib46] Zhang X., Xu H., Gong L., Wang J., Fu J., Lv Z., Zhou L., Li X., Liu Q., Xia P., Guo Y. (2024). Mannanase improves the growth performance of broilers by alleviating inflammation of the intestinal epithelium and improving intestinal microbiota. Anim Nutr.

